# Soil microbial communities associated with giant sequoia: How does the world's largest tree affect some of the world's smallest organisms?

**DOI:** 10.1002/ece3.6392

**Published:** 2020-06-12

**Authors:** Chelsea J. Carey, Sydney I. Glassman, Thomas D. Bruns, Emma L. Aronson, Stephen C. Hart

**Affiliations:** ^1^ Point Blue Conservation Science Petaluma CA USA; ^2^ Department of Microbiology and Plant Pathology University of California Riverside CA USA; ^3^ Department of Plant and Microbial Biology University of California Berkeley CA USA; ^4^ Department of Life and Environmental Sciences and the Sierra Nevada Research Institute University of California Merced CA USA

**Keywords:** bacteria, fungi, next‐generation amplicon sequencing, *Pinus lambertiana*, *Sequoiadendron giganteum*, Sierra Nevada

## Abstract

Giant sequoia (*Sequoiadendron giganteum*) is an iconic conifer that lives in relict populations on the western slopes of the California Sierra Nevada. In these settings, it is unusual among the dominant trees in that it associates with arbuscular mycorrhizal fungi rather than ectomycorrhizal fungi. However, it is unclear whether differences in microbial associations extend more broadly to nonmycorrhizal components of the soil microbial community. To address this question, we used next‐generation amplicon sequencing to characterize bacterial/archaeal and fungal microbiomes in bulk soil (0–5 cm) beneath giant sequoia and co‐occurring sugar pine (*Pinus lambertiana*) individuals. We did this across two groves with distinct parent material in Yosemite National Park, USA. We found tree‐associated differences were apparent despite a strong grove effect. Bacterial/archaeal richness was greater beneath giant sequoia than sugar pine, with a core community double the size. The tree species also harbored compositionally distinct fungal communities. This pattern depended on grove but was associated with a consistently elevated relative abundance of *Hygrocybe* species beneath giant sequoia. Compositional differences between host trees correlated with soil pH and soil moisture. We conclude that the effects of giant sequoia extend beyond mycorrhizal mutualists to include the broader community and that some but not all host tree differences are grove‐dependent.

## Graphical abstract

Giant sequoia (*Sequoiadendron giganteum*) is an iconic conifer that lives in relict populations on the western slopes of the California Sierra Nevada. In these settings, it is unusual among the dominant trees in that it associates with arbuscular mycorrhizal fungi rather than ectomycorrhizal fungi. We found that the effects of giant sequoia extend beyond mycorrhizal mutualists to include the broader community and that some but not all host tree differences are grove‐dependent.

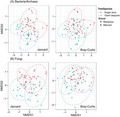

## 1 INTRODUCTION

There is increasing evidence that tree species influence a combination of soil chemical, physical, and biological properties (Hobbie et al., 2007; Mitchell, Campbell, Chapman, & Cameron, 2010; Zheng, Wei, & Zhang, 2017). For example, variation in litter chemistry, patterns of nutrient uptake, root exudation, and microclimate among tree species can alter rates of decomposition, soil nitrogen (N) and carbon (C) availability, and pH (Binkley & Giardina, 1998). Tree‐induced differences in resource availability and microclimate can, in turn, modify soil bacterial, archaeal, and fungal community composition (Glassman, Wang, & Bruns, 2017; Prescott & Grayston, 2013; Ushio, Wagai, Balser, & Kitayama, 2008) by encouraging microorganisms with compatible resource acquisition strategies and growth optima (Ayres et al., 2009). For example, Thoms, Gattinger, Jacob, Thomas, and Gleixner (2010) concluded that differences in soil pH beneath tree species in a temperate deciduous forest were partially responsible for observed differences in soil microbial communities. In addition, under some circumstances, direct association with host‐specific symbiotic microorganisms such as mycorrhizal fungi can further promote distinct soil fungal communities (Gao et al., 2013; Urbanová, Šnajdr, & Baldrian, 2015). Such plant‐induced changes to microbial communities can become more or less pronounced with time since plant establishment (Strayer, Eviner, Jeschke, & Pace, 2006), and can feed back on soil chemical and physical properties (Falkowski, Fenchel, & Delong, 2008), plant performance (Aponte, García, & Marañón, 2013; Bever et al., 2010), plant phenology (Wagner et al., 2014), and plant community composition (Van der Heijden, Bardgett, & Straalen, 2008).

Although soil microorganisms can directly and indirectly influence plant dynamics (Abbott et al., 2015; Reynolds, Packer, Bever, & Clay, 2003) and may mediate how plant communities respond to anthropogenic threats (Gehring, Sthultz, Flores‐Rentería, Whipple, & Whitham, 2017; Zolla, Badri, Bakker, Manter, & Vivanco, 2013), information on soil microbial communities associated with many rare or endemic tree species is limited. One such tree is the giant sequoia (*Sequoiadendron giganteum*)—a species that epitomizes charismatic megaflora (Hall, James, & Baird, 2011). Endemic to the western slope of the Sierra Nevada (California, USA), giant sequoia harbor a number of traits that make the species unique, ecologically interesting, and of conservation concern. Most conspicuously, giant sequoia are the world's largest trees, growing up to 87 m in height and reaching a bole volume of 1,500 m^3^ (Stephenson, 2000). They are also one of the longest‐lived trees, with an estimated average age of 2,230 years and a maximum known age of 3,266 years (Stephenson, 2000). Over the last century, fire suppression has threatened giant sequoia regeneration by minimizing canopy gaps and exposure of mineral soil, two important factors for germination of this shade intolerant tree (York, Battles, Eschtruth, & Schurr, 2011). Future conditions marked by increased and prolonged drought are expected to put additional stress on giant sequoia (Su et al., 2017). Fortunately, their charisma has deemed them one of the seven natural wonders of the United States (DeFries, 2013), and together, the ecological and cultural importance of the giant sequoia has promoted their current protection by both state and national agencies (Aune, 1994; Leisz, 1994).

Despite their iconic status, very little is known about how giant sequoia influence the soil and even less is known about how these titans of the tree world interact with some of the smallest yet most important organisms on the planet: microorganisms. The few prior studies that have been conducted suggest that, similar to other members of the *Cupressaceae* family (Alban, 1969), giant sequoia accumulate relatively large amounts of base cations such as calcium in their leaf litter resulting in high soil base saturation compared to other mixed‐conifer species (Zinke & Crocker, 1962; Zinke & Stangenberger, 1994). In those same studies, giant sequoia were also reported to maintain relatively high values of soil pH and soil organic matter. From a microbial perspective, giant sequoia are known to associate with arbuscular mycorrhizal fungi (AMF) (Fahey, York, & Pawlowska, 2012), symbiotic fungi that form relationships with the vast majority of herbaceous plants. This contrasts with most other trees in the Sierra Nevada, such as those of the *Pinaceae* and the *Fagaceae* families, whose woody roots instead associate with symbiotic ectomycorrhizal fungi (EMF) (Brundrett & Tedersoo, 2018). Few studies have probed the mycorrhizal dynamics of giant sequoia (Fahey et al., 2012; Molina, 1994), and none have intensively assessed soil bacterial/archaeal and fungal community structure (composition and diversity) using molecular techniques.

Given that giant sequoia occur on soils derived from various parent materials—including granite, diorite, and andesite bedrock—it is important to evaluate whether microbial communities beneath giant sequoia remain consistent or whether they vary across groves with different parent material. Parent material exerts a strong influence on soil properties especially in relatively undeveloped soils, and differences in underlying geology often interact with trees to shape soil microbial community structure (Carletti et al., 2009; Ulrich & Becker, 2006; Wagai, Kitayama, Satomura, Fujinuma, & Balser, 2011). For example, parent material and vegetation type interacted to affect soil macroaggregate size, and both factors also shaped microbial community structure following 30 years of surface exposure at reclaimed surface mining sites (Yarwood, Wick, Williams, & Daniels, 2015). The degree to which this occurs with the iconic giant sequoia, however, remains unknown.

We characterized bacterial/archaeal and fungal communities from beneath giant sequoia and a codominant ectomycorrhizal tree, sugar pine (*Pinus lambertiana*), through next‐generation amplicon sequencing of the 16S rRNA gene and the internal transcribed spacer region (ITS1). Sugar pine are prevalent on the western slope of the Sierra Nevada and are second only to giant sequoia in total volume, with individuals reaching 76 m in height and living up to 600 years (Hardin, Leopold, & White, 2000). We sampled soil from beneath 32 individuals of each tree species across two groves with contrasting geological substrates in Yosemite National Park, USA. By comparing these two tree species within and between groves, our experimental design allows us to evaluate for the first time the relative impact of these host trees and parent material on soil microbial structure. Specifically, we sought to address the following questions: (Q1) what bacterial, archaeal, and fungal members comprise the soil microbial community beneath giant sequoia individuals?; (Q2) how do these microbial communities compare to those beneath co‐occurring sugar pine individuals?; (Q3) are giant sequoia and sugar pine‐associated microbial communities consistent across groves with differing geological substrates?; and (Q4) which soil characteristics, if any, correlate with tree‐associated changes in microbial richness and composition?

## 2 METHODS

### 2.1 Site description

We sampled from two of the three giant sequoia groves contained within Yosemite National Park (YNP): Mariposa Grove (N 37.51539°, W 119.60435°) and Merced Grove (N 37.74982°, W 119.84061°). Both groves are located on the western slope of the Sierra Nevada in the rain–snow transition zone, and experience a Mediterranean‐type climate with warm–dry summers and cool–wet winters. In addition to giant sequoia and sugar pine, other common trees in these groves are the ectomycorrhizal trees white fir (*Abies concolor*) and ponderosa pine (*Pinus ponderosa*), and the arbuscular mycorrhizal tree incense cedar (*Calocedrus decurrens*) (Allen & Kitajima, 2014; Wang & Qiu, 2006). Both groves have relatively sparse understory vegetation composed primarily of AMF‐associated *Ceanothus* spp. and broadleaf lupine (*Lupinus latifolius*), as well seedlings and saplings of the EMF‐associated white fir (*Abies concolor*). Soils in the Mariposa Grove are derived from residuum and colluvium of metavolcanic (andesite and hornfels) with minor amounts of intermediate granitoid rock. Soils in the Merced Grove are derived from residuum and colluvium of quart‐rich metasedimentary rock (USDA NRCS, 2007). Despite contrasting parent materials, soils from both sites are classified within the same Soil Taxonomic Family: coarse–loamy, isotic, frigid Ultic Haploxeralfs. At the time of mineral soil sampling, O horizon thickness varied between 2.7 and 15.6 cm, and was greater in Merced than Mariposa Grove; within each grove, O horizon thickness was greater beneath giant sequoia than sugar pine trees (two‐way ANOVA: Tree, *p* = .027; Grove, *p* < .0001; Tree × Grove, *p* = .34).

### 2.2 Experimental design

Within each grove, we sampled from beneath 16 mature giant sequoia individuals and 16 co‐occurring mature sugar pine individuals. Because mature giant sequoia trees were generally the limiting experimental unit (especially in the Merced Grove), we first selected mature giant sequoia individuals whose crowns did not overlap with adjacent trees. We then selected the closest mature sugar pine trees to each giant sequoia individual that shared similar aspect, slope, landscape position, and understory species (when present). Relatively little understory vegetation occurred beneath each focal tree, and areas containing N‐fixing species such as *Ceanothus* spp. were avoided. As with giant sequoia, we ensured selection of sugar pine individuals whose crowns did not overlap with adjacent trees. This selection procedure was designed to minimize any confounding influences that may affect soil properties besides tree species. Our experimental design resulted in 32 total giant sequoia–sugar pine pairs with individuals that were located between 15 and 30 m of each other.

### 2.3 Soil sampling

In August 2013, we sampled bulk surface soil from beneath each tree individual (i.e., we did not directly target rhizosphere soil surrounding plant roots). We selected sampling locations midcrown and downslope of the tree bole assuming that aboveground litter would accumulate most at these locations, and therefore, the influence of trees would be maximal (Zinke & Crocker, 1962). Five replicate soil cores (0–5 cm depth of mineral soil) per tree were taken within an approximately 20 × 20 cm area using an Oakfield corer (1.9 cm diameter; Oakfield Apparatus Co) and composited into a single sample within a sterile plastic bag (Whirl‐Pak^®^, Nasco). The soil corer was sanitized after each composite sample using a rinse of 10% bleach followed by 95% ethanol. Soil samples were stored at 4°C, transported to University of California (UC) Merced, sieved (<2 mm; sanitized between samples as described above), and subsampled for microbial analysis. Additional soil subsamples were taken for 22 physicochemical analyses. These analyses were conducted on fresh or air‐dried soils, as appropriate for the assay (Methods S1).

### 2.4 DNA extraction

Each subsample was extracted immediately upon returning to the laboratory (within 24 hr), and two separate DNA aliquots per subsample were stored at −20°C for subsequent analysis. Specifically, we extracted microbial DNA from 0.250 g soil (±0.025) using a MO BIO PowerSoil Isolation Kit (Mo Bio Laboratories, Inc.) following the manufacturer's instructions. One 50 µl DNA aliquot was shipped on dry ice to UC Riverside for bacterial/archaeal analysis, and one was delivered on ice to UC Berkeley for fungal analysis.

### 2.5 16S rRNA amplicon preparation

After quantifying the extracted DNA using a NanoDrop 2000 (Thermo Fisher Scientific Inc.), we amplified each sample in duplicate using tailed primers targeting the V3–V4 region of the 16S rRNA gene (S‐D‐Bact‐0341‐b‐S‐17 and S‐D‐Bact‐0785‐a‐A‐21; Klindworth et al., 2013).We conducted polymerase chain reaction (PCR) by combining 2.5 μl DNA template, 5 μl each of 1 μM forward and reverse primers, and 12.5 μl KAPA HiFi HotStart ReadyMix (KAPA Biosystems, Inc.), totaling a 25 μl reaction. Thermal cycler conditions were the following: 95°C for 3 min., followed by 25 cycles of 95°C for 30 s, 55°C for 30 s, and 72°C for 30 s, followed by an extension step for 5 min. at 72°C. After amplification, we combined and purified the duplicate PCR products using Agencourt AMPure XP Beads (Beckman Coulter Genomics). A second round of PCR was subsequently conducted to attach dual indices to each sample using the Nextera XT Index Kit (Illumina Inc.). Briefly, 5 μl DNA, 5 μl each of 1 μM forward and reverse index primers, 25 μl KAPA HiFi HotStart ReadyMix, and 10 μl sterile water were combined to create a 50 μl mixture. Thermal cycler conditions were the following: 95°C for 3 min., followed by 8 cycles of 95°C for 30 s, 55°C for 30 s, and 72°C for 30 s, followed by an extension step for 5 min. at 72°C. We then conducted a second AMPure purification step on the indexed amplicons and quantified the products using the Quant‐iT™ PicoGreen^®^ dsDNA assay kit (Life Technologies Inc.). As a final step, we pooled the samples together in equimolar concentrations and sequenced them in one Illumina MiSeq PE 2x300 run at the UC Riverside Genomics Core Facility.

### 2.6 ITS1 amplicon preparation

We PCR amplified the ITS1 spacer from each sample using the ITS1F‐2 primer pair with Illumina MiSeq primers and single indexing designed by Smith and Peay (Smith & Peay, 2014). This primer pair is widely used in next‐generation amplicon sequencing and has a known specificity for basidiomycetes (Gardes & Bruns, 1993). We conducted PCR by combining 1 µl DNA template, 0.50 µl of 10 µM forward primer, 1 µl of the 10 µM bar‐coded reverse primer, 0.130 µl of HotStar Taq Plus (5 units/µl) DNA polymerase (Qiagen), 2.5 µl of 10× PCR buffer supplied by the manufacturer, and 0.50 µl 10mM each dNTPs; the 25 µl reaction was brought to volume with sterile water. Thermal cycler conditions were the following: 95°C for 5 min., followed by 29 amplification cycles of 94°C for 30 s, 51°C for 30 s, 72°C for 1 min., followed by a 10 min. final extension at 72°C. Amplifications for each indexed sample were cleaned with Agencourt AMPure XP Beads, quantified fluorescently with the Qubit dsDNA HS kit (Life Technologies, Inc.), pooled to equimolar concentration, and quality checked for amplicon size and concentration as described in Glassman, Levine, DiRocco, Battles, and Bruns (2016). The library was sequenced with Illumina MiSeq PE 2x250 at the Vincent J Coates Genomic Sequencing Laboratory, UC Berkeley.

### 2.7 16S sequence analysis

We obtained the 16S sequences already demultiplexed from the UC Riverside Genomics Core Facility and processed them using Quantitative Insights into Microbial Ecology (QIIME1) (Caporaso et al., 2010). After we joined the forward and reverse reads (allowing for 20% maximum difference within the region of overlap), we used default parameters to conduct quality control: reads were excluded if the length was less than 75 bases, if there were more than three consecutive low‐quality base calls, if less than 75% of the read length was consecutive high‐quality base calls, if a Phred score was below three, or if one or more ambiguous calls were present (Bokulich et al., 2013). After quality filtering, 3.9 M sequence reads remained.

Operational taxonomic units (OTU) with 97% similarity were picked using open reference UCLUST against the 13_8 release of the Greengenes database (DeSantis et al., 2006). Reads that did not match any sequences in the database were clustered de novo, and singletons were filtered out. Taxonomy was assigned in QIIME1 using the same Greengenes database. The median and mean number of sequences per samples were 60,494 and 61,422, respectively. After removing unassigned OTUs (1% of sequences), the OTU table was rarefied to 36,345 reads per sample, and as a result, one sugar pine sample was dropped.

### 2.8 ITS1 sequence analysis

We obtained the ITS sequences already demultiplexed from the UC Berkeley Vincent J Coates Genomic Sequencing Laboratory and processed them as in Glassman et al., (2016) using UPARSE (Edgar, 2013). We removed distal priming/adapter sites, trimmed the remaining untrimmed low‐quality regions from the ends, and then joined the forward and reverse reads. Paired reads were then quality filtered using the fastq_filter command in USEARCH and employing a maximum expected number of errors of 0.25, which is a strict error criterion ensuring high‐quality reads. After quality filtering, 2.9 M sequence read pairs remained. We picked OTUs at 97% similarity, then reference‐based chimera detection was employed using USEARCH and referencing against the UNITE database accessed on 10.09.2014 (Kõljalg et al., 2005). We assigned taxonomy in QIIME1 using the same UNITE database. The resulting OTU table yielded 2,173 total OTUs. The median and mean number of sequences per samples were 41,801 and 39,946, respectively. Only OTUs that were identified to the Kingdom Fungi were retained, and after removing all OTUs with a No Blast Hit, the OTU table was rarefied to 13,904 reads per sample; as a result, one giant sequoia and two sugar pine samples were dropped.

### 2.9 Statistical analysis

We used a multifaceted approach to assess microbial community structure of giant sequoia soils and to compare the structure of these communities with those beneath sugar pine. We first tested the main and interactive effects of plant species and grove on bacterial/archaeal and fungal OTU richness (alpha diversity) by performing a two‐way analysis of variance (ANOVA), transforming the data for normality and homogeneity of variance when necessary. We then visualized similarities in microbial community composition between tree species and grove using nonmetric multidimensional scaling (NMDS) of the Jaccard (presence–absence) and Bray–Curtis (relative abundance) dissimilarity metrics. To determine whether beta diversity differed significantly between tree species and grove, we performed the multivariate permutation test perMANOVA using the "adonis" function in the R VEGAN package (permutations = 999) (Oksanen et al., 2018). Because an assumption of perMANOVA is equal variance between groups, we also performed an analysis of multivariate homogeneity of group dispersions (permDISP; Table 1).

**TABLE 1:** Results of the perMANOVA and permDISP for (A) bacteria/archaea and (B) fungi

	PERMANOVA			PERMDISP
	*F*_model_	*R* ^2^	*p* value	*p* value
**(A) Bacteria/Archaea**				
*Jaccard*				
Site	3.66	0.057	.001	.50
Tree Species	2.29	0.036	.001	.88
Site × Tree	1.16	0.018	.088	
*Bray–Curtis*				
Site	9.43	0.134	.001	.28
Tree Species	4.09	0.063	.001	.78
Site × Tree	1.30	0.017	.145	
**(B) Fungi**				
*Jaccard*				
Site	4.06	0.064	.001	.19
Tree Species	2.32	0.038	.001	.07
Site × Tree	1.34	0.021	.016	
Tree Species in Merced Grove	1.98	0.062	.001	.98
Tree Species in Mariposa Grove	1.83	0.064	.002	.01
*Bray–Curtis*				
Site	3.59	0.047	.001	.03
Tree Species	1.62	0.027	.007	.03
Site × Tree	1.38	0.022	.032	
Tree Species in Merced Grove	1.79	0.056	.001	.02
Tree Species in Mariposa Grove	1.26	0.045	.096	.16

Note

Results are presented for both Jaccard and Bray–Curtis dissimilarity matrices. Because there was a significant tree × site interaction for fungi, we present the effects of tree species separated by grove. Site = Mariposa versus Merced Grove. Tree Species = giant sequoia versus sugar pine.

As a complement to these multivariate tests, we compared the relative abundance of bacterial/archaeal and fungal phyla within each grove using nonparametric Mann–Whitney *U* test on ranks. In addition, we analyzed the frequency and relative abundance of dominant OTUs across species and grove, given that abundant taxa tend to contribute significantly to ecosystem functioning (Dai et al., 2016). OTU dominance thresholds have historically been defined arbitrarily, often set at an average relative abundance of 1% (Dai et al., 2016). However, in our study, only one bacterial OTU met this criterion, and rank abundance characteristics differed markedly between bacteria/archaea and fungi. We therefore designated the top 20 most relatively abundant OTUs (averaged across all samples in each dataset) as “abundant.” For each abundant OTU, we examined the frequency across samples and determined whether there were significant differences in relative abundance by tree species within each grove using nonparametric Mann–Whitney *U* tests.

Because organisms that consistently associate with a particular host or habitat likely play a critical functional role (Shade & Handelsman, 2012), we identified OTUs that were found to ubiquitously associate with either giant sequoia or sugar pine trees across both groves. This enabled us to capture and identify the microbial members that are core to each tree species. To determine the core OTUs, we first identified OTUs occurring in 100% of the samples from each tree‐grove dataset (giant sequoia in Merced Grove; giant sequoia in Mariposa Grove; sugar pine in Merced Grove; sugar pine in Mariposa Grove) using the compute_core_microbiome.py script in QIIME1. We then compared the four lists of OTUs and graphically represented the overlap using Venn diagrams. For giant sequoia, we focused our analysis and discussion on the core OTUs that were not also ubiquitous in sugar pine, and vice versa. These core bacterial/archaeal OTUs were summarized at the phylum level, and we determined the taxonomy of those OTUs that were also more frequent (>20% difference) beneath giant sequoia than sugar pine using EzTaxon (Kim et al., 2012). The presence/absence of these OTUs from all samples were illustrated graphically with the heatmap3 package (Zhao, Guo, Sheng, & Shyr, 2014). We provide no such summary for fungi, as neither giant sequoia nor sugar pine had a core microbiome based on our 100% occurrence definition. Even when the definition of a core was relaxed to 80% frequency, giant sequoia soils contained only two core fungal OTUs and sugar pine soils contained one.

Finally, we ran separate analyses for two subsets of fungal taxa that included only EMF or AMF. This allowed us to determine whether these root symbionts differed significantly by host tree and grove. EMF taxa were bioinformatically parsed as previously established (Glassman et al., 2016), and AMF were bioinformatically parsed to include only individuals of the Glomeromycota phylum (Redecker & Raab, 2006). While AMF‐specific small subunit (SSU) marker gene sequencing is the preferred approach to characterize AMF, general fungal primers like those used here are typically sufficient for generating comparative estimates of community structure and identifying responses to environmental variables (Lekberg et al., 2018). We compared the richness and relative abundance of EMF and AMF by host tree and grove using 2‐way ANOVA, visualized Jaccard dissimilarity using NMDS plots, and analyzed differences by group using perMANOVA in the same ways as described above.

**FIGURE 1:**
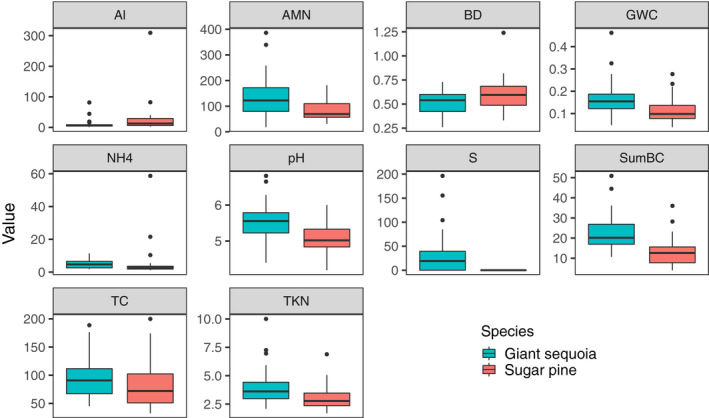
Boxplots of selected physicochemical properties in 0–5 cm mineral soil under giant sequoia and sugar pine trees across both groves. All of the displayed soil properties differed significantly between tree species, and significant interactions with grove generally did not occur. NH_4_^+^ concentrations could not be transformed for normality and were therefore analyzed using nonparametric Mann–Whitney *U* test on ranks; all other data were analyzed using a two‐way ANOVA (*α* = .05). Note different *Y*‐axis scales. Al = extractable aluminum (mg/kg); AMN = anaerobic mineralizable nitrogen (mg/kg); BD = bulk density (Mg/m^3^); GWC = gravimetric water content (kg/kg); NH_4_ = ammonium concentrations (mg/kg); pH = –log [H^+^]; S = extractable sulfur (mg/kg); SumBC = sum of base cations (cmolc/kg); TC = total carbon (g/kg); TKN = total Kjeldahl nitrogen (g/kg)

In addition to determining differences in microbial community structure, we aimed to identify whether any structural differences may be attributed to tree‐induced changes in soil parameters. To that end, univariate Spearman rank correlations were conducted to determine whether any of the measured physicochemical parameters correlated with microbial richness. We used Spearman rank correlations as a conservative estimate because in many cases the data were not normally distributed. In addition, we used simple and partial Mantel tests to examine correlations between each physicochemical parameter and microbial community composition (Mantel, 1967). We performed the Mantel tests using Jaccard measures of dissimilarity for microbial composition and Euclidean distances for each soil parameter. The partial Mantel tests controlled for the effects of all the other soil parameters while assessing the relationship between the Jaccard dissimilarity matrix and the focal soil parameter. Because we were particularly interested in understanding if and how giant sequoia influence microbial communities via changes in soil properties, we only included physicochemical parameters that were found to differ significantly by tree species in our analyses (Figure 1; Figure S1)—namely soil pH, bulk density, anaerobically mineralizable nitrogen (AMN), NH_4_^+^ concentrations, total C, total N, extractable sulfur (S), extractable aluminum (Al), sum of the base cations (composed of 76%–91% calcium, 5%–16% magnesium, 2%–11% potassium, and 0%–3% sodium), and gravimetric soil moisture. We conducted separate analyses for each grove to avoid capturing microbial–soil relationships that may be due to a grove rather than a host tree effect. For all analyses, *α* = .05 was used to denote statistical significance. Analyses were conducted in R version 3.2.1 (R Core Team, 2017).

## 3 RESULTS

### 3.1 Characterizing Giant Sequoia Bacterial/Archaeal and Fungal Communities (Q1)

Across both the Mariposa and Merced groves, Proteobacteria comprised the majority of the 16S sequences recovered beneath giant sequoia (31.5% ± SE 0.5%), followed by Acidobacteria (15.9% ± SE 0.5%), Actinobacteria (15.6% ± SE 0.4%), Planctomycetes (11.2% ± SE 0.3%), and Verrucomicrobia (9.0% ± SE 0.2%; Figure S2). Together, these five phyla accounted for greater than 80% of the sequences. Forty‐one less abundant phyla were also recovered from giant sequoia soils, three of which were archaeal.

Across both the Mariposa and Merced groves, Basidiomycota comprised the majority of ITS1 sequences recovered beneath giant sequoia (82.1% ± SE 2.4%), followed by Ascomycota (12.5% ± SE 1.5%), and Zygomycota (2.1% ± SE 1.4%). Less than 2% were Glomeromycota or unidentified fungi (Figure S2). *Hygrocybe* was the most dominant Basidiomycota genus recovered*,* comprising 21.5% (± SE 5.8%) of all sequences. *Wilcoxina* was the most relatively abundant Ascomycota genus recovered from beneath giant sequoia, comprising 4.9% (± SE 1.1%) of all sequences.

### 3.2 Comparing giant sequoia and sugar pine microbial communities across both groves: Alpha and beta diversity (Q2 and Q3)

**FIGURE 2:**
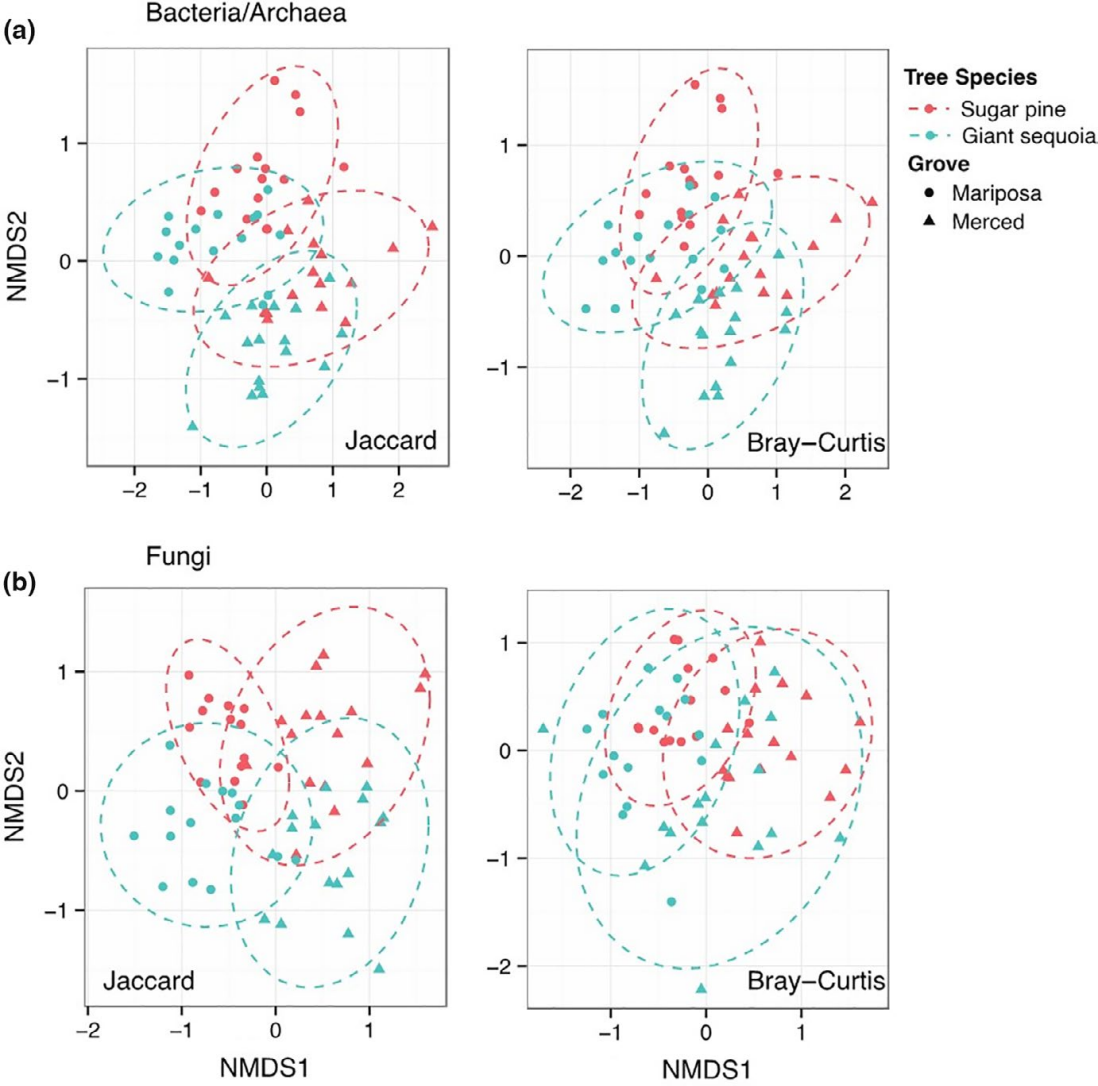
Influence of tree species and grove on (a) bacterial/archaeal and (b) fungal community composition. Left panel = nonmetric multidimensional scaling (NMDS) of Jaccard (presence/absence) dissimilarity metric. Right panel = NMDS of Bray–Curtis (relative abundance) dissimilarity metric. Each symbol corresponds to a sample collected from one of two groves, and each color corresponds to a tree species. Points that are close together represent samples with similar community composition, and the dashed ovals represent 95% confidence intervals of sample ordination grouped by unique tree × grove combinations. The stress values for the bacterial/archeal ordinations were 0.06 (Jaccard) and 0.07 (Bray–Curtis); the stress values for the fungal ordinations were 0.14 (Jaccard) and 0.18 (Bray–Curtis)

**FIGURE 3:**
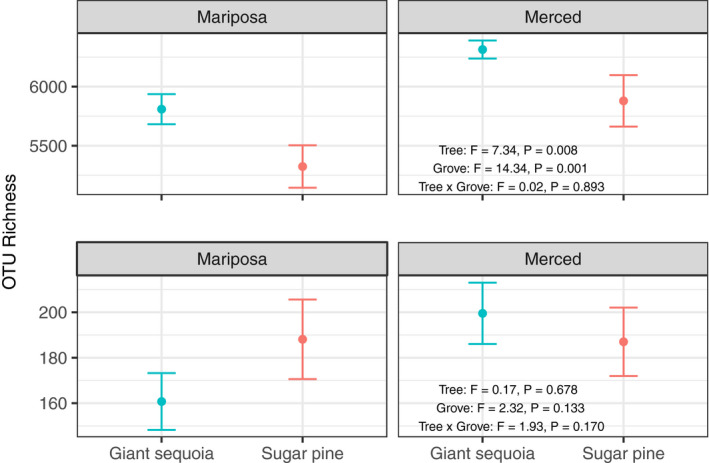
Influence of tree species and grove on alpha diversity for bacteria/archaea (top panels) and fungi (bottom panels). OTU Richness = number of observed operational taxonomic units, presented as mean ± 1 *SE*. *F*‐ and *p*‐values were derived from a two‐way ANOVA

Bacterial/archaeal communities were most strongly structured by grove effects, followed by host tree differences (Figure 2, Table 1). In addition, bacterial/archaeal richness was greater under giant sequoia (mean richness ± SE, Mariposa Grove: 5,809.3 ± 127.5, Merced Grove: 6,314.2 ± 76.5) compared to sugar pine (mean richness ± SE, Mariposa Grove: 5,323.8 ± 179.6, Merced Grove: 5,879.5 ± 217.9; Tree Species, *p* = .008; Grove, *p* = .001; Figure 3). These differences remained constant across grove (no significant Tree × Grove interaction, *p* = .89). At the phylum level, Proteobacteria, Actinobacteria, and Gemmatimonadetes were relatively more abundant—and Acidobacteria, Armatimonadetes, and TM7 were relatively less abundant—in giant sequoia compared to sugar pine soils (Figure S2). However, these differences were not consistent across groves and some were only marginally significant (*p* = .05–0.10).

Fungal communities were also structured most strongly by grove effects, followed by host tree differences (Figure 2; Table 1). However, in contrast to bacteria/archaea, there was a significant tree by grove interaction (Tree × Grove interaction, Jaccard: *p* = .016; Bray–Curtis: *p* = .032), and fungal richness did not significantly differ between tree species (giant sequoia mean richness ± SE, Mariposa Grove: 160.7 ± 12.5, Merced Grove: 199.5 ± 13.5; sugar pine mean richness ± SE, Mariposa Grove: 188.1 ± 17.5, Merced Grove: 187.0 ± 15.1; Tree Species, *p* = .68; Grove, *p* = .13; Tree × Grove interaction, *p* = .17; Figure 3). At the phylum level, Basidiomycota and Glomeromycota were relatively more abundant—and Ascomycota and Zygomycota were relatively less abundant—in giant sequoia compared to sugar pine soils (Figure S2). These phylum‐level differences were not consistent across groves, and some were marginally significant (*p* = .05–0.1).

The composition of EMF communities differed by host tree and grove with no interaction, and AMF communities differed by host tree but not grove (Figure S3). Host tree differences in AMF were at least partially driven by a dispersion effect (PERMDISP, *p* = .03). EMF had greater relative abundance beneath sugar pine than giant sequoia (*p* = .002), and AMF had greater relative abundance beneath giant sequoia than sugar pine (*p* = .02; Figure S4). AMF communities, but not EMF communities, showed a significant host tree effect for OTU richness, with AMF communities beneath giant sequoia harboring significantly more OTUs than those beneath sugar pine (giant sequoia mean richness ± SE = 8.5 ± 1.09; sugar pine mean richness = 5.4 ± 0.96; *p* = .008).

### 3.3 Comparing giant sequoia and sugar pine microbial communities across both groves: Dominant taxa and core members (Q2 and Q3)

**FIGURE 4:**
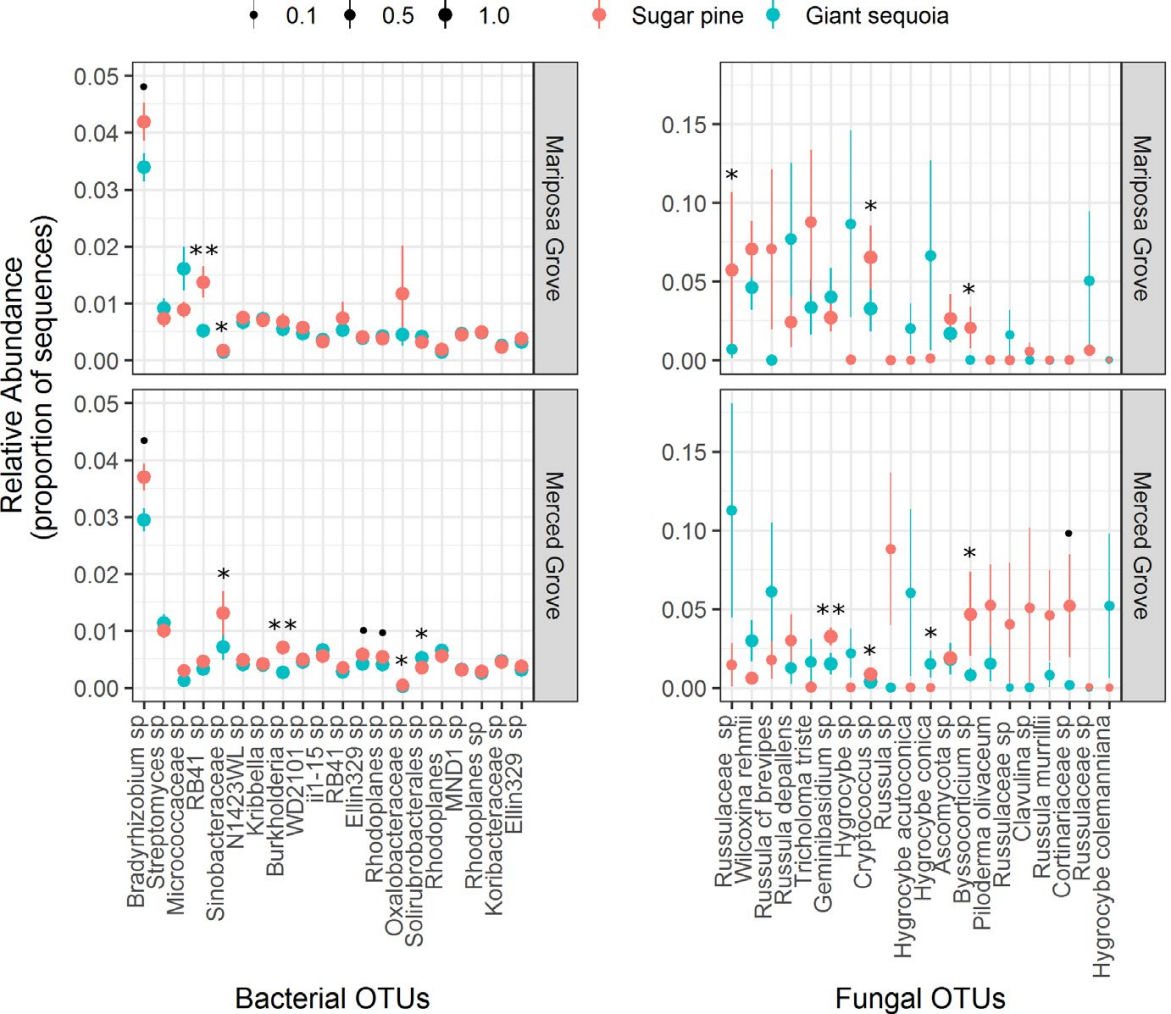
The relative abundance of the 20 most abundant bacterial/archaeal and fungal OTUs in giant sequoia and sugar pine soils across both groves. Error bars = 1 *SE* of the mean. The size of each point is scaled by the frequency of an OTU (how many samples it was recovered from), with larger circles corresponding to greater frequency. Significant differences in OTU relative abundance between tree species were assessed using Mann–Whitney *U* test on ranks (^•^ *p* < .1, *p < .05, **p < .01)

The 20 most abundant bacterial/archaeal OTUs had average relative abundances that ranged from 0.34% to 3.5% of the sequences in a given sample. Of these OTUs, 50% were Proteobacteria and the most abundant OTU was a *Bradyrhizobium* species (Figure 4). The 20 most abundant fungal OTUs had higher average relative abundances than their bacterial/archaeal counterparts, ranging from 1.4% to 4.8% of the sequences in a given sample. Sixty‐five percent of these fungal OTUs were EMF and 20% belonged to the genus *Hygrocybe*. We observed a number of dominant bacterial and fungal OTUs whose frequency, relative abundance, or both differed consistently between tree species. For example, in both Mariposa and Merced groves, the *Bradyrhizobium* sp. and *Sinobacteraceae* sp. were relatively more abundant beneath sugar pine (Figure 4; Table S1). In addition, an unidentified *Cryptococcus* species was recovered from 100% of samples across both groves, but was consistently more relatively abundant beneath sugar pine (Figure 4). An unidentified *Byssocorticium* species, an EMF taxon, also showed a consistent trend across groves, where it was more frequent and relatively abundant beneath sugar pine compared to giant sequoia. In contrast, species of *Hygrocybe*, which are generally considered to be saprotrophic (although see discussion below), were almost always more frequent and relatively more abundant in giant sequoia soils, although these differences were not always statistically significant (Figure 4; Table S1). Finally, some OTUs differed significantly between tree species in one grove but not the other. This included an unidentified *Russulaceae* sp., another EMF taxon (Tedersoo, May, & Smith, 2010), which was more frequent and relatively abundant beneath sugar pine than giant sequoia in the Mariposa grove—and an unidentified *Geminibasidium* sp., which is a xerotolerant basidiomycete yeast (Nguyen, Nickerson, & Seifert, 2013), was relatively more abundant beneath sugar pine than giant sequoia in the Merced grove.

**FIGURE 5:**
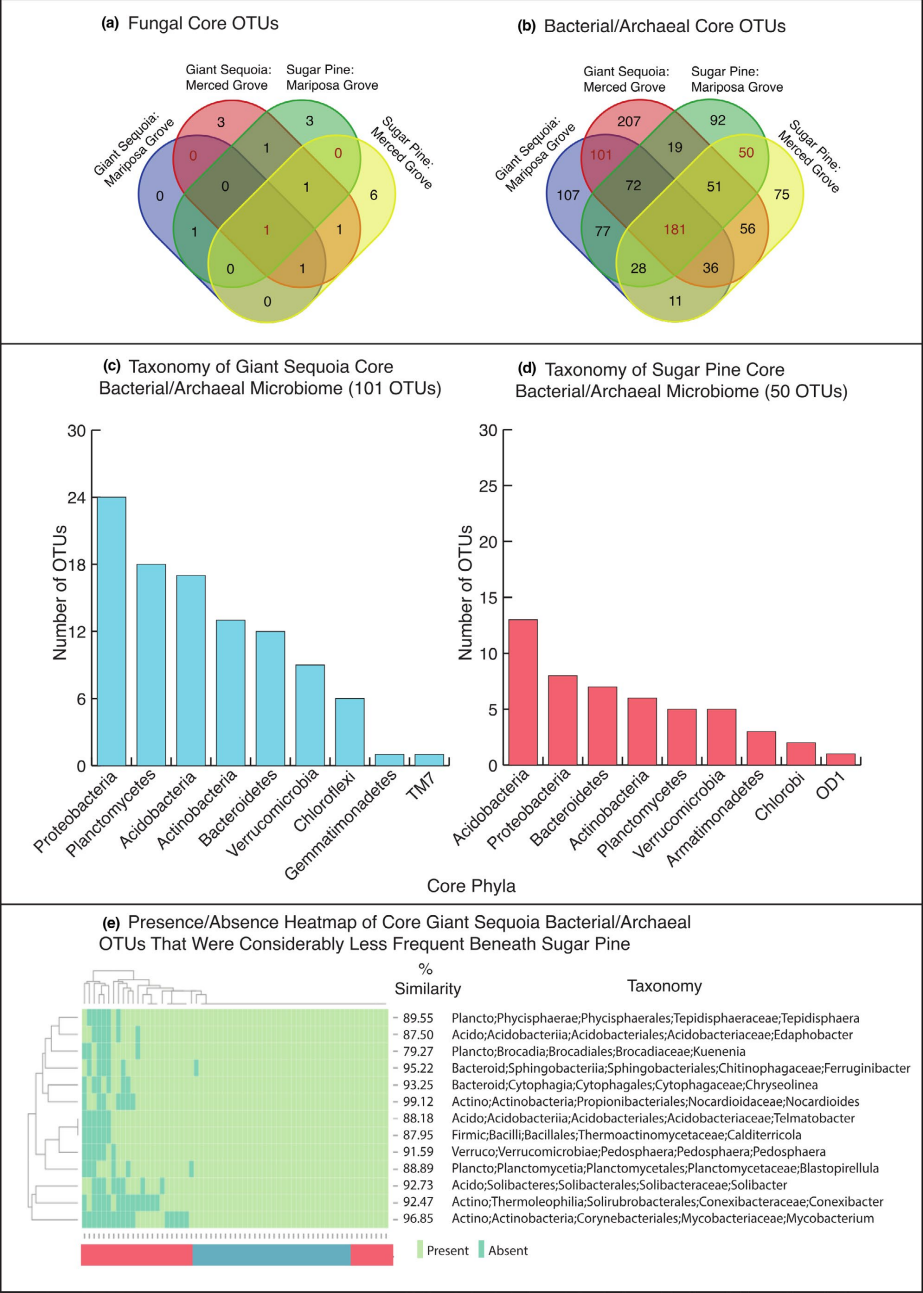
Shared OTUs of the (a) fungal and (b) bacterial/archaeal core microbiomes associated with giant sequoia and sugar pine. The venn diagrams show absolute number of OTUs shared between core microbiomes of each tree species across two groves. Phylum‐level taxonomic information is also provided for the OTUs comprising the (c) giant sequoia and (d) sugar pine core bacterial/archaeal communities. (e) Heatmap illustrating presence/absence and taxonomy of core OTUs of giant sequoia that were considerably less frequent (20% difference) in sugar pine soils. Taxonomic information was derived from EzTaxon, and % similarity is the sequence similarity between the OTU and its nearest cultured match. Colors of the bar beneath the heatmap correspond to tree type (red = sugar pine, blue = giant sequoia)

There were very few fungal OTUs that were recovered from 100% of giant sequoia or sugar pine samples in either grove. Accordingly, neither giant sequoia nor sugar pine had a core fungal microbiome comprised of OTUs that were recovered from all samples (Figure 5). Even when we relaxed the definition of a core to require only 80% frequency, giant sequoia soils contained only two core fungal OTUs and sugar pine soils contained one—both of which were Zygomycota. In contrast, bacterial/archaeal communities beneath giant sequoia and sugar pine harbored a number of OTUs that comprised a core (Figure 5). Both tree species contained the phyla Proteobacteria, Acidobacteria, Planctomycetes, Actinobacteria, Bacteroidetes, and Verrucomicrobia in their core community. Each core also contained a number of OTUs representing phyla unique to that tree species. For instance, giant sequoia contained core members from Chloroflexi, Gemmatimonadetes, and TM7, while sugar pine did not. Similarly, only sugar pine contained core members from Armatimonadetes, Chlorobi, and OD1 (Figure 5). In addition to these phylum‐level differences, the size of the core for giant sequoia and sugar pine differed, with giant sequoia containing substantially more core OTUs (101 OTUs) than sugar pine (50 OTUs). However, of the 101 core OTUs beneath giant sequoia, only 13% were notably less frequent (frequency < 80%) in sugar pine soils (Figure 5; Table S2). Similarly, of the 50 core OTUs beneath sugar pine, only 10% were notably less frequent in giant sequoia soils (Table S2). In all other cases, OTUs that comprised the core of one tree community were often missing from only a few samples in the other tree community (80%–95% frequency).

### 3.4 Determining which soil characteristics, if any, correlate with tree‐associated changes in microbial richness and composition (Q4)

Giant sequoia and sugar pine soils differed significantly in a number of measured physicochemical parameters. Specifically, giant sequoia soils had lower bulk density values and extractable aluminum concentrations—in addition to higher total C, total N, ammonium concentrations, anaerobically mineralizable N, soil pH, sum of base cations, extractable S, and soil moisture—than sugar pine soils (Figure 1). Of these parameters, bacterial/archaeal and fungal community composition correlated most strongly and consistently with soil pH (Table 2); these relationships with soil pH remained strong even when the effects of all other soil parameters were statistically controlled for using a partial Mantel test. The composition of bacterial/archaeal and fungal communities also tended to be strongly related to differences in soil moisture in both the simple and partial Mantel tests. In addition, bacterial/archaeal and fungal community composition correlated with extractable Al and the sum of base cations; however, these relationships were inconsistent between groves for fungi and often disappeared for both microbial groups when the effects of other soil parameters were accounted for (Table 2).

**TABLE 2:** Correlations between (A & B) soil parameters and microbial composition, and (C) soil parameters and richness

(A) Microbial composition—Mantel				
Soil property	Bacteria/Archaea		Fungi	
	Mariposa	Merced	Mariposa	Merced
Bulk Density	0.02	0.05	0.13	−0.02
Soil Moisture	0.23*	0.20	0.28*	0.27*
pH	0.57**	0.80**	0.32**	0.44**
Al	0.43**	0.58**	0.05	0.33**
SumBC	0.22*	0.27*	0.17	0.29**
S	0.11	0.04	0.08	0.17
AMN	0.12	0.06	0.05	0.20
NH_4_^+^	0.01	0.02	0.06	−0.02
Total Nitrogen	0.01	−0.02	0.08	0.14
Total Carbon	−0.02	−0.06	0.06	−0.04

(B) Microbial composition—partial Mantel				
Soil Property	Bacteria/Archaea		Fungi	
	Mariposa	Merced	Mariposa	Merced
Bulk Density	−0.05	0.05	0.11	0.02
Soil Moisture	0.28*	0.32*	0.33*	0.10
pH	0.50**	0.77**	0.32*	0.35**
Al	0.36*	−0.05	−0.04	0.13
SumBC	0.09	0.18*	−0.02	0.04
S	−0.08	−0.23^•^	−0.06	0.01
AMN	0.01	0.04	−0.17^•^	0.09
NH_4_^+^	−0.01	0.12	0.03	0.02
Total Nitrogen	−0.03	−0.27*	0.00	−0.06
Total Carbon	−0.07	0.00	−0.02	−0.04

(C) Microbial richness—Spearman's correlation				
Soil property	Bacteria/Archaea		Fungi	
	Mariposa	Merced	Mariposa	Merced
Bulk Density	0.23	0.20	0.46*	0.08
Soil Moisture	0.21	−0.33^•^	−0.13	−0.08
pH	0.06	0.57**	−0.28	0.10
Al	−0.06	−0.58**	0.26	−0.09
SumBC	0.16	0.19	−0.16	0.20
S	0.14	0.22	−0.03	0.07
AMN	0.30	−0.02	−0.08	0.13
NH_4_^+^	0.09	−0.14	−0.10	0.05
Total Nitrogen	0.15	−0.08	−0.05	0.10
Total Carbon	0.20	−0.26	0.06	0.05

Note

Soil moisture = gravimetric water content; Al = extractable aluminum; SumBC = sum base cations; S = sulfur; AMN = anaerobically mineralizable nitrogen.

* *p* < .05

** *p* < .01

• *p* = .05–.10.

In contrast to microbial composition, bacterial/archaeal and fungal richness were largely unrelated to the measured physicochemical parameters (Table 2). Only in the Merced Grove did bacterial/archaeal richness correlate positively with pH (Figure S5), and negatively with extractable aluminum. Similarly, of all the physicochemical parameters, fungal richness only correlated (positively) with bulk density in the Mariposa Grove.

## 4 DISCUSSION

Giant sequoia are the largest, and some of the longest‐lived, trees in the world. An iconic species with great ecological and cultural importance, little is known about how giant sequoia individuals interact with belowground communities. In this study, we used next‐generation amplicon sequencing to describe for the first time the soil microbiome associated with this charismatic megaflora. We compared giant sequoia microbiomes to those of co‐occurring sugar pine trees across two groves on contrasting geological substrates in Yosemite National Park, USA, and determined whether host tree differences in microbial communities were related to differences in soil physicochemical parameters. While this kind of observational study design limits our power of causal inference, it is a useful way to explore this undercharacterized system and generate hypotheses that can be used to guide future microbiome research on the giant sequoia.

### 4.1 Characterizing giant sequoia soil microbiomes and comparing them to sugar pine microbiomes across both groves (Q1–Q3)

Differences in bacterial/archaeal community composition between groves tended to be greater than those differences associated with tree species. Still, there was evidence that giant sequoia influenced underlying bacteria and archaea in unique ways compared to sugar pine. Specifically, we found that bacterial and archaeal richness was greater beneath giant sequoia than sugar pine (Figure 3), possibly because giant sequoia are larger and create more niche space within the soil (Glassman, Lubetkin, Chung, & Bruns, 2017). However, differences in physicochemical properties may also play a role (see discussion below). In addition, communities of bacteria/archaea were compositionally distinct from those beneath sugar pine (Figure 2). These differences remained constant across the two groves despite the fact that soils from each grove were derived from geologically distinct substrates. Fungal community composition also differed between giant sequoia and sugar pine; however, the specific ways that fungi differed between tree species depended on the grove (Figure 2). Overall, these findings—which agree with a number of other studies where bulk soil microbial communities differed between contrasting tree species (Ayres et al., 2009; Scheibe et al., 2015; Sun et al., 2016; Thoms et al., 2010)—suggest that a “host signal” can still be observed despite relatively large differences by grove.

Giant sequoia and sugar pine are known to associate with two contrasting groups of mycorrhizal fungi. The former with AMF (Fahey et al., 2012) and the latter with EMF (Walker, 2001). AMF and EMF were recovered from beneath both host trees—possibly because of understory influences or root overlap. However, mycorrhizal communities associated with giant sequoia differed from those associated with sugar pine (Figures S3 and S4). AMF communities were relatively more abundant and more diverse beneath giant sequoia. In contrast, EMF communities were relatively more abundant beneath sugar pine, with EMF taxa such as an unidentified *Byssocorticium* species tending to be more frequent and relatively abundant beneath this host tree species. While this is to be expected, our findings show that differences in mycorrhizal communities in mixed stands of AMF and EMF trees can be seen at the bulk soil scale, which can have cascading effects on biogeochemical processes including the cycling of C (Averill, Dietze, & Bhatnagar, 2018) and N (Mushinski et al., 2019).

Microbial taxa that are abundant within the community can contribute significantly to ecosystem function (Dai et al., 2016). To discern patterns in abundant OTUs between trees, we filtered both the bacterial/archaeal and fungal OTU tables to include only the 20 most relatively abundant OTUs. In accordance with the idea that microbial communities often include a very small number of dominant members and are instead comprised of many rare members (Lynch & Neufeld, 2015), one bacterial OTU, which was of the genus *Bradyrhizobium*, averaged 3.5% relative abundance across all samples, making it by far the most dominant bacterial OTU in both groves (Figure 4). Recent work employing quantitative population genomics suggests that free‐living *Bradyrhizobia* are unexpectedly incapable of fixing atmospheric N and may instead metabolize aromatic C sources (VanInsberghe et al., 2015), which may explain why this genus tends to dominate microbial communities of forest bulk soil (Hartmann et al., 2012; Uroz, Buée, Murat, Frey‐Klett, & Martin, 2010; VanInsberghe et al., 2015). The unidentified *Bradyrhizobium* OTU had greater relative abundance in sugar pine compared to giant sequoia soils—although this difference was only marginally significant. In addition to having greater relative abundance of the *Bradyrhizobium* OTU, sugar pine soils also had higher relative abundances of other OTUs in the Proteobacteria phylum, including an unidentified OTU in the *Sinobacteraceae* family*—*some members of which have the capacity to degrade cellulose and lignin (Wilhelm, Singh, Eltis, & Mohn, 2019).

In contrast to bacteria/archaea, the rank abundance curve for the 20 most dominant fungal OTUs had a shallow gradient (i.e., the average relative abundances were more even; Figure 4). While the majority of these OTUs were EMF, 20% of them were from the genus *Hygrocybe* (waxcaps)*. Hygrocybe* are widespread and can be found in a variety of habitats worldwide (Halbwachs, Karasch, & Griffith, 2013), including forests of the *Sequoiadendron* sister genus *Sequoia* (Glassman, personal observation). These fungi are frequently considered to be saprotrophic; however, recent work has implicated some *Hygrocybe* species, such as *H. virginea*, as being endophytic (Halbwachs et al., 2013; Tello et al., 2014), and highly elevated δ^15^N values of Hygrophoraceae basidiocarps suggest that *Hygrocybe* may acquire their N from unusual sources like soil invertebrates (Halbwachs et al., 2018). In our study, *Hygrocybe* were rarely recovered from beneath sugar pine, corroborating previous assertions that species in this genus avoid EMF‐dominated habitats (Halbwachs et al., 2013). While the exact trophic lifestyle of this genus remains uncertain, it is possible that—given the preponderance of *Hygrocybe* species in giant sequoia soils and their apparent ability to act as root and systemic endophytes—these fungi may form ecologically significant symbiotic relationships with giant sequoia (Zarraonaindia et al., 2015). Future research should therefore identify whether such a relationship exists and, if so, what the ecological implications may be for giant sequoia growth and survival.

In addition to identifying common and abundant OTUs, it can be useful to distinguish core members of a microbial community that remain constant across space or time (Shade & Handelsman, 2012). Doing so helps define a healthy (or alternatively a degraded) community, and can improve our understanding of how that community will respond to future perturbations. In contrast to fungi, which had no discernable core community, we identified considerable core bacterial/archaeal communities associated with both tree species (Figure 5). Interestingly, the size of the bacterial/archaeal core associated with giant sequoia was double that of sugar pine, indicating that this giant, long‐lived tree maintains a relatively large and consistent set of bacterial/archaeal OTUs in its surrounding soil. The larger community associated with giant sequoia could be due to its age and size, as larger trees are known to host more microbial taxa (Glassman, Lubetkin, et al., 2017).

Thirteen of these core bacterial/archaeal OTUs were also considerably (at least 20%) less frequent in sugar pine soils. However, there were no clear trends in the taxonomy or ecology of these thirteen OTUs, with family associations ranging from *Nocardioidaceae* (contains endophytes and species capable of degrading organic matter; Tóth & Borsodi, 2014) to *Mycobacteriaceae* (contains animal pathogens and species capable of degrading hydrocarbons; Lory, 2014). Metagenomic data could provide a more complete picture of core community dynamics, as some evidence suggests that communities assemble at the functional rather than the phylogenetic level (Burke, Steinberg, Rusch, Kjelleberg, & Thomas, 2011). Regardless, our data contribute to a small set of previously published work that explicitly identify core communities in bulk soil (Andrew et al., 2012; Orgiazzi et al., 2013), and indicate that giant sequoia and sugar pine each harbor core communities of bacteria/archaea, but lack consistent core fungal OTUs at the spatial scale studied here.

It is possible that having a large and diverse core community of bacteria/archaea aids in the long‐term success of giant sequoia individuals. In addition to providing critical biogeochemical functions (e.g., decomposition of organic matter and nutrient cycling), core and abundant microorganisms within soil may act as a source that “seeds” rhizospheric and endospheric communities, ultimately contributing directly to plant health. Indeed, it has been proposed that some foliar endophytes persist through the winter as saprobes of litter only to reinvade host leaves in the spring (Baldrian, 2017; Unterseher, Peršoh, & Schnittler, 2013). A recent study assessing foliar bacterial endophytic communities of giant sequoia and coastal redwoods (*Sequoia sempervirens*) found that giant sequoia contained a diverse endophytic community, with major phyla including Acidobacteria, Actinobacteria, Bacteroidetes, Firmicutes, Fusobacteria, Proteobacteria, and TM7 (Carrell & Frank, 2015). Notably, five of twenty dominant orders recovered from giant sequoia foliage samples were represented in our core bacterial/archaeal community dataset (Actinomycetales, Burkholderiales, Rhizobiales, Rhodospirillales, and Sphingobacteriales). It is conceivable that at least some of these endophytes are derived from the soil community; however, (at minimum) comparative sampling of giant sequoia microbial communities within the same site, and (at maximum) use of more advanced tracing techniques (e.g., isotopes or quantum dots), will be required to assess this. Future studies that focus on connecting the giant sequoia holobiont with soil communities should provide promising insights into the stability of this tree species over millennia.

### 4.2 Determining which soil characteristics, if any, correlate with tree‐associated changes in microbial richness and composition (Q4)

Trees can affect the composition and diversity of soil microbial communities through a variety of indirect mechanisms. Some of these mechanisms include changes in root exudation, nutrient uptake, soil microclimate, pH, and the amount and quality of litter inputs (Binkley & Giardina, 1998). In our study, ten measured soil parameters differed between giant sequoia and sugar pine (Figure 1), and microbial community composition correlated with a number of them. Most strongly and consistently, bacterial/archaeal and fungal community composition related to soil pH and soil moisture (Table 2). Soil moisture dynamics are coupled to water stress, oxygen diffusion, and substrate supply; differences in soil moisture can therefore alter metabolic activity of microbial functional groups, the occurrence of aerobic/anaerobic processes (e.g., aerobic decomposition/denitrification), and ultimately microbial community composition (Docherty et al., 2015; Manzoni, Schimel, & Porporato, 2012). Soil pH is a well‐known driver of bacterial/archaeal community composition and diversity, a driver that is often unrivaled by other soil parameters due to the narrow tolerance range of these organisms (Collins, Carey, Aronson, Kopp, & Diez, 2016; Lauber, Hamady, Knight, & Fierer, 2009). While fungi are thought to exhibit wider pH ranges for optimal growth (Rousk et al., 2010), recent studies indicate that soil pH can also be a strong mediator of fungal community composition—a finding consistent with ours (Glassman, Wang, et al., 2017; Siles & Margesin, 2016).

Although we found community composition to correlate with soil pH, only in one case (bacterial/archaeal richness in the Merced Grove) did soil pH correlate positively and significantly with OTU richness. The significant relationship in the Merced Grove appears to be driven by a few samples that had pH levels below 4.7 (Figure S5). In a continental‐scale study across North and South America, soil pH and bacterial diversity were shown to demonstrate a unimodal rather than linear relationship, leveling out between pH 6.0 and 6.5 and then declining thereafter (Lauber et al., 2009). While other explanations may exist, it is possible that differences in the pH range between Merced (pH 4.19–6.25) and Mariposa Groves (4.83–6.81), while small, played a role in the former but not the latter showing a significant relationship between bacterial/archaeal richness and this predictor variable.

There is growing recognition that calcium availability also helps to explain variation in soil microbial community composition within and across sites (Allison, Yermakov, Miller, Jastrow, & Matamala, 2007; Docherty et al., 2015; Lladó, López‐Mondéjar, & Baldrian, 2018). Calcium provides structure to many plant cell walls, and the cycling of this cation in soil depends largely on litter decomposition (Chapin, Matson, & Vitousek, 2011). In our study, giant sequoia litter was significantly enriched in base cations compared to sugar pine, and this resulted in increased extractable calcium levels (in addition to Mg^2+^, K^+^, Na^+^) in the upper mineral soil. Mantel tests revealed that bacterial/archaeal and fungal community composition correlated significantly with the sum of the base cations, a composite measure that was composed primarily of calcium. However, this relationship almost always disappeared when the effects of the other variables were accounted for (Table 2), indicating that correlations between microbial composition and sum of the base cations were generally spurious. As others have suggested (Lladó et al., 2018), it is likely that base cations, and calcium in particular, indirectly influenced microbial composition in these groves via effects on soil pH. Although, it is also possible that calcium may influence the soil community via effects on other properties like soil aggregation (Allison et al., 2007; Rowley, Grand, & Verrecchia, 2018).

Taken together, these data suggest that giant sequoia indirectly influence the composition of underlying microbial communities in part by maintaining high soil pH and moisture content compared to sugar pine. The long‐lived nature of the host trees makes it likely we are detecting tree‐induced effects; however, the possibility that giant sequoia and sugar pine preferentially establish on microsites with contrasting soil and microbial characteristics to begin with cannot be ruled out. Characterizing giant sequoia microbiomes as a function of time since establishment across additional groves with (dis)similar parent materials will help to disentangle the causal relationship.

### 4.3 Conclusion

Using next‐generation amplicon sequencing, we show for the first time that microbial communities of bulk soil differ between giant sequoia and co‐occurring sugar pine. Namely, the soil bacterial/archaeal and fungal communities of giant sequoia were compositionally distinct from sugar pine, with greater bacterial/archaeal richness and a larger core community. These host tree differences, which were at least partially related to soil pH and moisture, were discernible despite concurrently large grove effects. In some cases, the influence of host tree differed between the two groves under study, which were close in proximity but had contrasting parent material. While the degree to which these patterns occur across giant sequoia's larger geographic range is an open question, our findings suggest that the effects of giant sequoia extend beyond mycorrhizal mutualists to include the broader community at the bulk soil scale.

## CONFLICT OF INTEREST

The authors declare that they have no conflict of interest.

## AUTHOR CONTRIBUTION

**Chelsea J. Carey:** Conceptualization (supporting); data curation (lead); formal analysis (lead); investigation (lead); methodology (lead); project administration (lead); validation (lead); visualization (lead); writing – original draft (lead); writing – review & editing (lead). **Sydney I. Glassman:** Data curation (supporting); formal analysis (supporting); methodology (supporting); writing – review & editing (supporting). **Thomas D. Bruns:** Funding acquisition (supporting); resources (supporting); writing – review & editing (supporting). **Emma L. Aronson:** Funding acquisition (supporting); resources (supporting); supervision (supporting); writing – review & editing (supporting). **Stephen C. Hart:** Conceptualization (lead);data curation (supporting); funding acquisition (supporting); methodology (supporting); project administration (supporting); resources (supporting); supervision (supporting); writing – review & editing (supporting).

## Supporting information

Figures S1‐S5

Table S1

Table S2

Methods S1

## Data Availability

All raw 16S and ITS sequences have been deposited to the NCBI Sequence Read Archive under BioProject PRJNA580285. Relevant physicochemical parameters have been deposited to Dryad (https://doi.org/10.6071/M3WH4T).
