# Supplementary material for: Soil microbial communities associated with giant sequoia: How does the world's largest tree affect some of the world's smallest organisms?
Source: Ecol Evol. 2020 Jun 12;10(13):6593–609. doi: 10.1002/ece3.6392 (PMC7381575; doi:10.1002/ece3.6392)
Supplement: Supplementary file 1 — Figures S1‐S5 [file ECE3-10-6593-s001.docx]

**Figure S1.** Spearman’s rank correlations between soil variables separated by grove (Top = Merced Grove; Bottom = Mariposa Grove). The upper triangle shows the correlation coefficients and associated significance, while the lower triangle illustrates the nature of the relationship between variables. The diagonal shows the distribution of data for each parameter. The size of the text on the upper triangle is a function of the correlation strength, with larger text denoting stronger correlations. * P < 0.05, ** P < 0.01, *** P < 0.001. Al = extractable aluminum (mg/kg); AMN = anaerobically mineralizable nitrogen (mg/kg); BD = bulk density (Mg/m^3^); GWC = gravimetric water content (kg/kg); NH4 = ammonium concentrations (mg/kg); pH = -log [H^+^]; S = extractable sulfur (mg/kg); SumBC = sum of base cations (cmolc/kg); TotalC = total carbon (g/kg); TKN = total Kjeldahl nitrogen (g/kg).


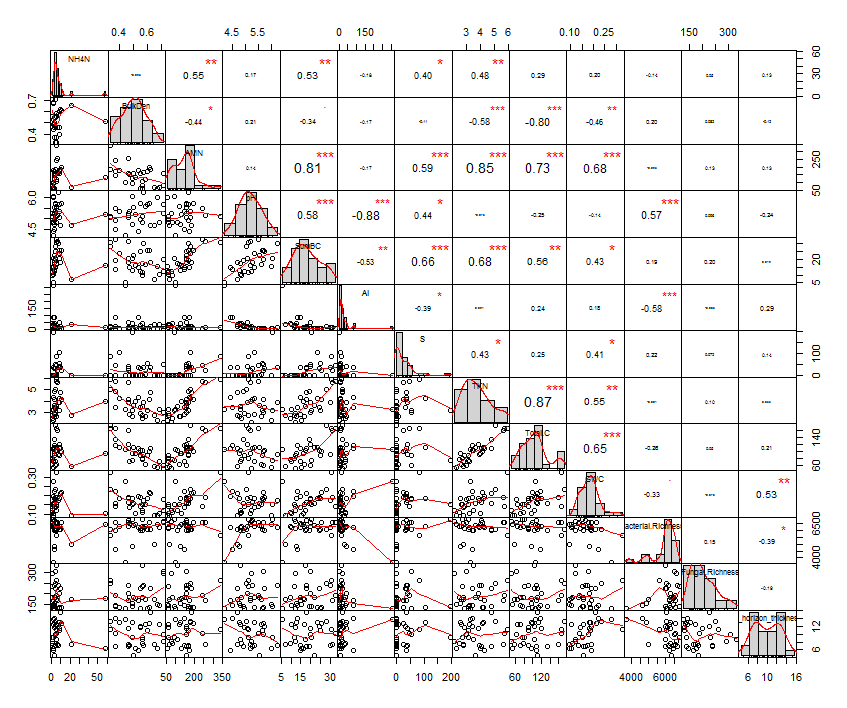


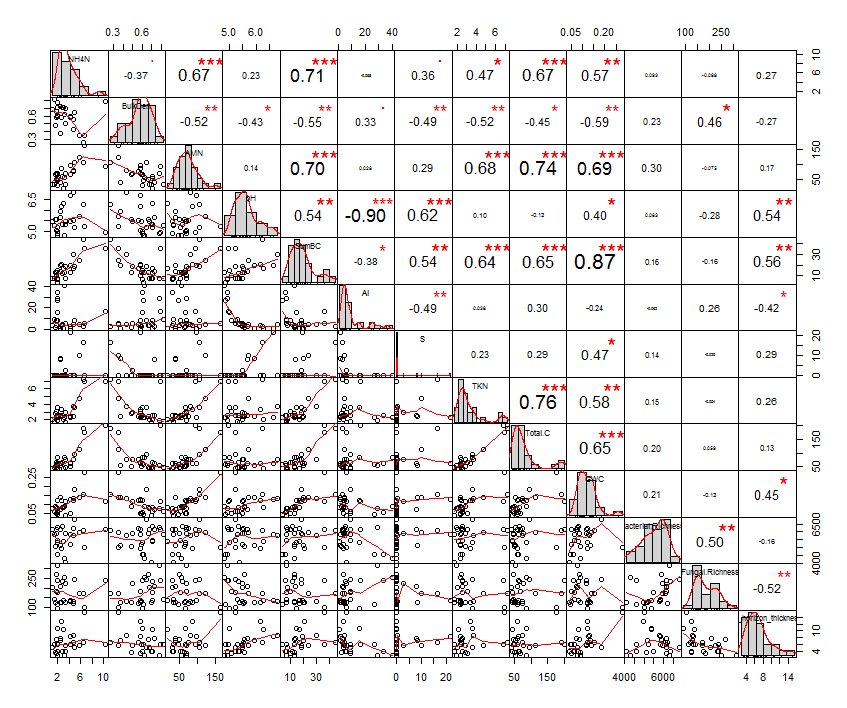


**Figure S2.** Relative abundances of (A) bacterial and archaeal phyla and (B) fungal phyla averaged across replicates from beneath giant sequoia and sugar pine individuals in Mariposa and Merced groves. Both graph and legend share the same order, sequential from bottom to top. For bacteria/archaea, ‘other’ indicates the combined relative abundance of 26 lesser abundant phyla. Significant (P < 0.05) and marginally significant (P = 0.05-0.10) differences in phyla between tree species are illustrated for each grove (derived from Mann-Whitney U test on ranks).

**Figure S3.** Influence of tree species (giant sequoia and sugar pine) and grove (Mariposa and Merced Grove) on (left) ectomycorrhizal (EMF) and (right) arbuscular mycorrhizal (AMF) community composition. Figures are non-metric multidimensional scaling (NMDS) of Jaccard (presence/absence) dissimilarity metric. Each color corresponds to a sample collected from a particular tree by grove combination. Points that are close together represent samples with similar community composition. perMANOVA and PERMDISP results also shown from Jaccard dissimilarity metric for both EMF and AMF. The stress values for EMF and AMF fungi were 0.21 and 0.14, respectively.


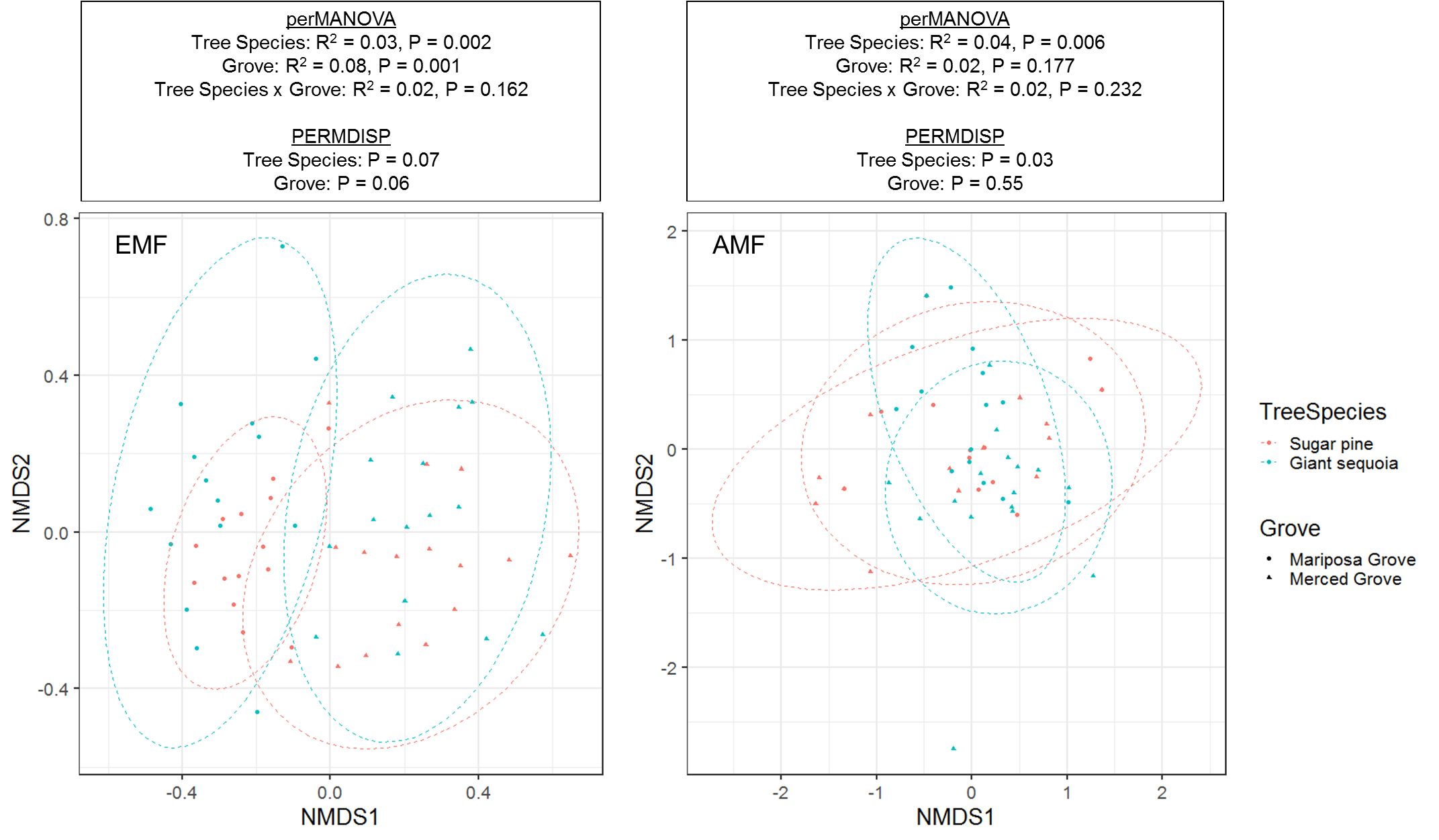


**Figure S4.** Boxplots showing the variation in relative abundance (top panels) and OTU richness (bottom panels) of ectomycorrhizal fungi (EMF) and arbuscular mycorrhizal fungi (AMF) by tree species and grove. The table displays two-way ANOVA results, which were used to assess the effects of tree species and grove on each response variable.

**
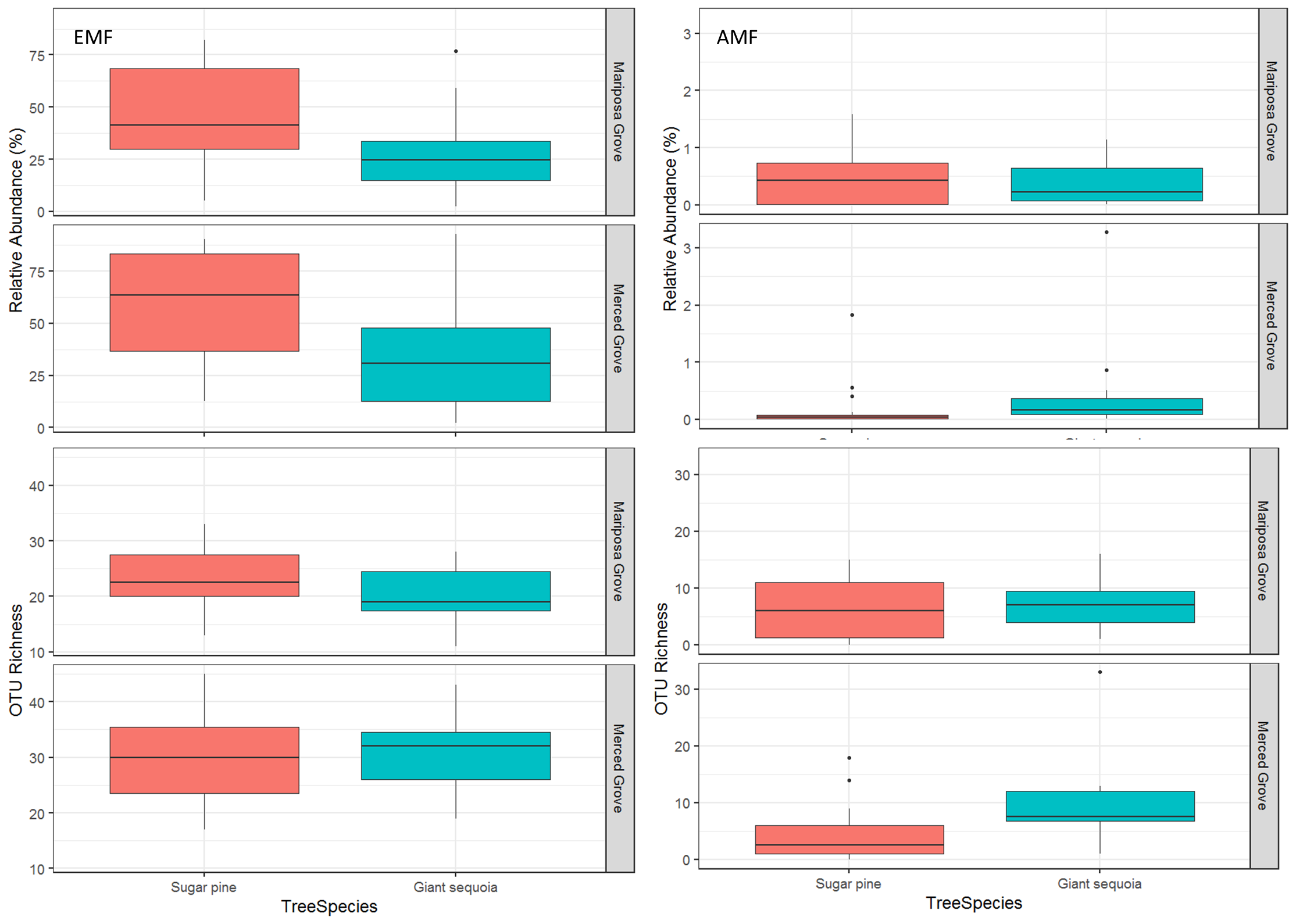
**

|  | EMF  Relative Abundance | EMF  OTU Richness | AMF  Relative Abundance | AMF  OTU Richness |
| --- | --- | --- | --- | --- |
| Tree Species | F = 10.73,  P = 0.002 | F = 0.52,  P = 0.48 | F = 5.36,  P = 0.02 | F = 7.47,  P = 0.008 |
| Grove | F = 3.66,  P = 0.06 | F = 22.75,  P < 0.001 | F = 2.29,  P = 0.14 | F = 0.002,  P = 0.97 |
| Tree Species x Grove | F = 0.19,  P =0.67 | F = 0.85,  P = 0.36 | F = 0.68,  P = 0.41 | F = 1.04,  P = 0.31 |

**Figure S5.** Bacterial/archaeal OTU richness as a function of soil pH for Merced Grove (left) and Mariposa Grove (right). Blue points = giant sequoia, red points = sugar pine.

**
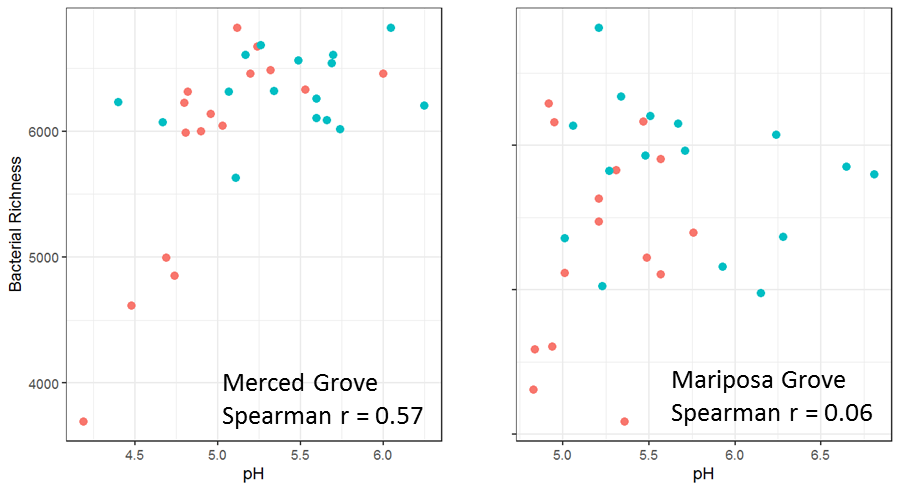
**
