# Supplementary material for: Soil microbial communities associated with giant sequoia: How does the world's largest tree affect some of the world's smallest organisms?
Source: Ecol Evol. 2020 Jun 12;10(13):6593–609. doi: 10.1002/ece3.6392 (PMC7381575; doi:10.1002/ece3.6392)
Supplement: Supplementary file 4 — Methods S1 [file ECE3-10-6593-s004.docx]

**Methods S1. Physicochemical Analyses**

Gravimetric water content was determined by oven-drying a 15-mL subsample of sieved soil for 48 h at 105 °C. Soil pH was measured using an Orion Ross Sure-flow combination epoxy body electrode and an Orion Star ATC probe connected to an Orion Dual Star pH/ISE benchtop meter (Thermo Scientific, Waltham, MA, USA). The suspensions (0.01 M CaCl_2_ solution at a 1:5 soil mass:solution ratio) were mixed thoroughly with a mechanical stirrer and then allowed to equilibrate for 30 minutes before measurement. Ammonium and NO_3_^-^ concentrations were determined by extracting 5 g of field-moist soil with 25 mL of 2 M potassium chloride (KCl), filtered through Whatman No. 1 filter paper (preleached with deionized water), followed by flow injection analysis (QuikChem 8500, Lachat Instruments, Hach Company, Loveland, CO). Ammonium and NO_3_^–^ were determined colorimetrically (alkaline-phenolate and sulfanilamide/N-(1-Naphthyl)ethylenediamine cadmium reduction methods, respectively). To determine anaerobically mineralizable N, at the time of KCl-extraction, an additional 15-mL subsample was placed in a 120 mL specimen cup and 50 mL of deionized water was added. The suspension was mixed gently and then incubated at 40 °C for 7 days. After this period, 50 mL of 4 M KCl were added and the sample was shaken, filtered, and analyzed as above. Anaerobically mineralizable N was calculated as the difference in NH_4_^+^ concentrations between incubated and the initial unincubated samples. Available P was measured on air-dried soil using a Bray-1 extraction. Twenty-five mL of a mixed, 0.03 M NH_4_F + 0.025 M HCl solution were added to ≈2.5 g of air-dried soil in a 50 mL polyethylene centrifuge tube and shaken for 5 min at 150 cycles per minute. The suspension was then filtered through a quantitative filter paper and stored at < 4 °C until analysis using an ascorbic acid colorimetric method. Total Kjeldahl N and P were determined using the Kjeldahl digestion protocol on air-dry, finely ground subsamples. After digestion, samples were analyzed by flow-injection colorimetry using salicylate and molybdate-ascorbic acid methods, respectively. Total C was determined by EA-IRMS at UC Davis’ Stable Isotope Facility. Extractable elements (Na, K, Mg, Ca, Al, Fe, and S) in soil were measured adding 25 mL of 1 M NH_4_Cl to ≈2.5 g of air-dried soil in a 50 mL polyethylene centrifuge tube. The suspension was shaken for 30 min at 150 cycles per minute and then filtered through a quantitative filter paper and store at < 4 °C until analysis. Solutions were diluted 1:10 (v/v) with deionized water containing 1% HNO_3_ prior to analysis by ICP-AES.
